# In Silico and In Vitro Profiling of Honokiol and Paclitaxel-Loaded PBM Nanoparticles for Targeted Breast Cancer Delivery

**DOI:** 10.3390/ph18121814

**Published:** 2025-11-27

**Authors:** Briana Kinnel, Amit Kumar Srivastava, Santosh Kumar Singh, Rajesh Singh

**Affiliations:** 1 Department of Microbiology, Biochemistry and Immunology, Morehouse School of Medicine, Atlanta, GA 30310, USAsksingh@msm.edu (S.K.S.); 2 School of Forensic Sciences, Uttar Pradesh State Institute of Forensic Sciences, Lucknow 22604, India; 3 Cancer Health Equity Institute, Morehouse School of Medicine, Atlanta, GA 30310, USA

**Keywords:** nanoparticle, paclitaxel, honokiol, MUC1, breast cancer, drug delivery system

## Abstract

**Background/Objectives:** This study aimed to further enhance the properties of paclitaxel (PTX) and honokiol (HNK) through encapsulation in planetary ball-milled nanoparticles (PBM NPs) and specific targeting of breast cancer (BrCa) cells via MUC1 targeting using an aptamer (S2.2) coating. **Methods:** Tissue microarray (TMA) analysis was utilized to measure MUC1 expression in stages 1, 2, 3, and 4 BrCa tissue samples. Pharmacokinetic simulations were performed to explore the potential advantages of using PTX and HNK in combination while targeting MUC1 for BrCa treatment. To investigate the efficacy of the PBM NPs for MUC1 targeting, we synthesized the aptamer-conjugated PTX and HNK PBM NPs (PTX-S2.2-PBM NPs, HNK-S2.2-PBM NPs) using N-hydroxysuccinimide (NHS) coupling. Dynamic light scattering (DLS), Fourier-transform infrared (FTIR), and high-performance liquid chromatography (HPLC) were utilized to characterize the NPs. MTT and live/dead cell assays were used to evaluate the cytotoxicity of the NPs. **Results:** TMA sample analysis confirmed the upregulation of MUC1 in BrCa tissues, which increased with the stage of BrCa. DLS analysis revealed that the PTX-S2.2 and HNK-S2.2 NPs have a desirable size (83.4 nm and 163 nm, respectively) and zeta potential (−9.74 mV and −7.16 mV, respectively), which are suitable for systemic circulation and improved therapeutic outcomes. FTIR and HPLC analysis suggest proper conjugation was achieved, and an encapsulation efficiency of PTXS2.2 and HNKS2.2 NPs at 77% and 84%, respectively, was achieved. Cell viability assays demonstrated that PTX-S2.2-PBM and HNK-S2.2-PBM NPs exhibit cytotoxicity comparable to or greater than free PTX and HNK, respectively. **Conclusions:** These findings support the belief that using PTX-S2.2 and HNK-S2.2 PBM NPs could be a promising treatment option for BrCa.

## 1. Introduction

Despite many developments in cancer treatment approaches, breast cancer (BrCa) is still the leading cause of cancer-related death in women [1]. Paclitaxel (PTX) is a chemotherapeutic agent commonly used as a first-line treatment for BrCa [2]. Though highly efficacious, PTX is known to have many side effects due to its toxicity. Combination therapy, which utilizes both chemotherapy and a nontoxic natural compound, has been demonstrated to reduce chemotherapy toxicity to normal cells and enhance efficacy compared to monotherapies [3]. Honokiol (HNK), a natural compound isolated from Magnolia grandiflora, exhibits potent anticancer properties, including the induction of apoptosis, inhibition of metastasis, and reversal of multiple drug resistance (MDR) [3]. The co-encapsulation of PTX and HNK to treat BrCa has been shown to have a synergistic effect, enhancing the cytotoxicity of PTX [4].

The use of targeted nano-therapies to treat BrCa has also become a promising treatment approach. The selective binding and uptake of targeted drug delivery systems into cancer cells has increased drug efficacy, enhanced distribution, and reduced toxicity compared to traditional nonspecific cancer treatments [5]. There are various types of nano-therapies; however, they all have disadvantages, such as poor aqueous solubility, bioavailability, and absorption [6]. Planetary ball milled nanoparticles (PBM NPs), on the other hand, utilize a starch core and biodegradable copolymers, are uniformly milled into spherical shape and size, have been shown to have loading efficiency of both hydrophilic and hydrophobic drugs up to >90%, and have a controlled surface logP for systemic, oral, or cutaneous delivery [7]. PBM NPs can be formulated with copolymer surface coatings, such as polycaprolactone (PCL) and polyethylene glycol (PEG), as well as ligands, like aptamers, that target overexpressed receptors, enabling targeted drug delivery. McFadden et al. demonstrated that using PTX and fisetin PBM NPs conjugated with folic acid (FA) resulted in an increased occurrence of apoptosis and inhibition of cell proliferation [8]. Similarly, Singh et al. demonstrated that FA-conjugated resveratrol and docetaxel-loaded PBM NPs caused a significant increase in the number of apoptotic cells by 30.92% and 65.9% when treating prostate cancer cells [9].

The oncogene Mucin 1 (MUC1) belongs to the mucin family [10]. MUC1 is overexpressed in many epithelial adenocarcinomas, such as lung, liver, colon, breast, pancreas, and ovarian cancers [11]. MUC1 upregulation is linked to increased aggressiveness, invasion, and chemoresistance [12,13]. In normal cells, MUC1 is found on the apical surface, plays a role in anti-cell adhesion, and serves as a protective barrier, hydrant, and lubricant. MUC1 plays a significant role in the mammary ducts in the milk fat globule membranes [14]. However, the extracellular domain in carcinomas is often hypoglycosylated, and cell polarity is lost due to MUC1′s altered spatial distribution [15,16]. The hypoglycosylation of MUC1 has been shown to stimulate endocytosis and the intracellular accumulation of MUC1 [17]. A study investigating MUC1 expression in 52 cases of early-stage triple-negative breast cancer (TNBC) found that 94% of the cases were MUC1-positive, with 67% exhibiting moderate to strong MUC1 expression, 27% demonstrating weak MUC1 expression, and 6% lacking MUC1 expression [18]. Due to MUC1′s differential expression in cancers and its association with cancer development and progression, it has been identified as a viable therapeutic target for BrCa [19].

Using aptamers as a targeting agent has many benefits, including high specificity and affinity for tumor targeting, versatility, enhanced stability and immunogenicity, improved drug delivery, and reduced side effects [20,21]. Research has shown that the MUC1 aptamer S2.2 exhibits a high affinity and specificity for binding to MUC1 [22]. Yu et al. utilized S2.2 for the targeted delivery of PTX-loaded poly (lactic-co-glycolic-acid) (PLGA) nanoparticles in MCF-7 BrCa [23]. It was found that S2.2 conjugation enhanced in vitro drug delivery and cytotoxicity in MUC1 overexpressing cancer cells when compared to nonspecific NPs. Unlike prior uses of MUC1 targeting in BrCa, our current study utilized a unique carrier system, PBM NPs, that are coated with starch/PCL/PEG, to investigate the potential of PTX and HNK co-delivery. Starch has been shown to be a biodegradable, biocompatible natural polymer that can assist in the controlled release of encapsulated drugs [24]. Similarly, the use of biocompatible and biodegradable co-polymers PCL/PEG in NP synthesis can enhance circulation, increase drug bioavailability, prolong blood circulation, and enable targeted ligand binding [25]. The present study employed both in vitro and in silico methods to explore the efficacy of PTX and HNK encapsulation in BrCa-specific PBM NPs, PTX-S2.2-NPs, and HNK-S2.2-NPs, respectively, in (TNBC) cell lines. Our findings will potentially extend this strategy to treat TNBC, which is known to have very limited treatment options.

## 2. Results

### 2.1. MUC1 Expression in Human BrCa Tissues

**Figure 1:**
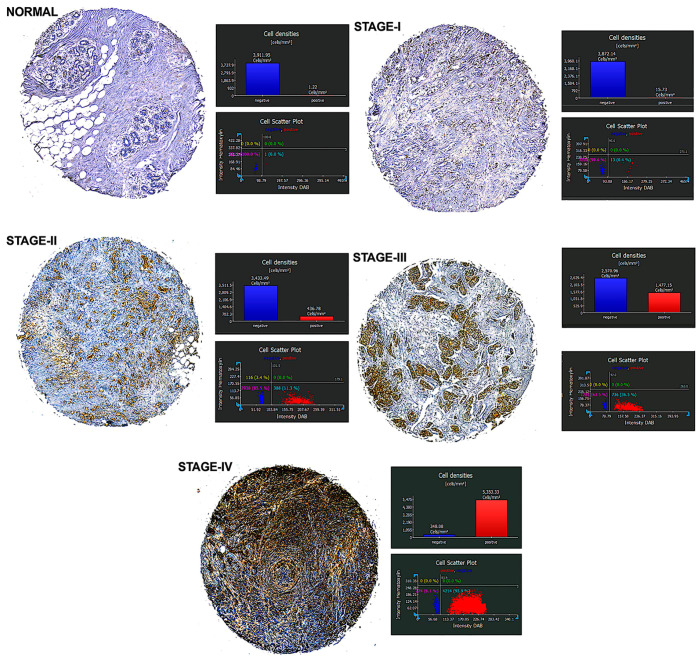
Expression of MUC-1 in Breast tissues. Tissues from normal breast and stages I, II, III, and IV breast cancer were stained with MUC-1 antibodies—representative cases showing immune intensities of MUC-1 (diaminobenzidine (DAB), brown).

We investigated the expression of MUC1 protein in human breast cancer tissues using immunostaining with an anti-hMUC1 monoclonal antibody and compared it with MUC1 expression in normal breast tissues. MUC1 expression was detected in most breast cancer tissues, while no expression was detected in normal breast tissues. Analysis of BrCa specimens indicated that MUC1 expression is directly related to the stage of BrCa. As the stage of cancer increased, MUC1-positive expression increased significantly, with the highest MUC1 expression observed in Stage IV BrCa tissue (93.9%) compared to Stage III (36.5%), Stage II (11.3%), and Stage I (0.4%) (Figure 1). These findings suggest that MUC1 targeting may be a viable treatment option for patients with BrCa, particularly those with late-stage disease.

### 2.2. Functionalization and Characterization of PBM NPs

#### 2.2.1. Size and Zeta Potential of PBM NPs

**Figure 2:**
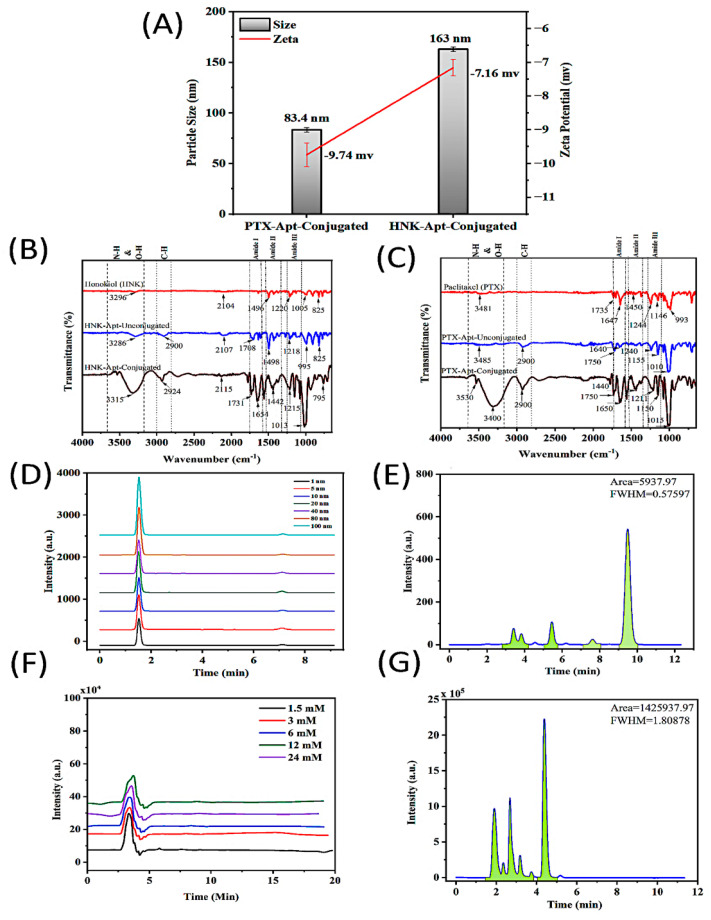
Characterization of breast cancer cell-specific PBM nanoparticles for charge, size, surface chemistry, and encapsulation efficiency. MUC1 aptamer S2.2 conjugated PCL-PEG coated and uncoated nanoparticles (**A**) size and zeta potential measured using a Malvern Zeta Sizer NS instrument (Malvern Panalytical, Westborough, MA, USA). (**B**) Fourier-transform infrared spectrum of the synthesized aptamer conjugated Paclitaxel (PTX) PBM nanoparticle, unconjugated PTX PBM nanoparticle, and free PTX. (**C**) Fourier-transform infrared spectrum of synthesized aptamer conjugated honokiol (HNK) PBM nanoparticle, unconjugated HNK PBM nanoparticle, and free HNK. The data were collected in absorption mode with 64 background scans and the wave number ranging from 4000 to 500 cm^−1^. HPLC analysis of standard concentrations of Free HNK (**D**) and aptamer-conjugated HNK (**E**) and free PTX (**F**) and aptamer-conjugated PTX (**G**).

Size and zeta potential are essential properties of NPs that can significantly impact bioavailability and cellular uptake. To verify that the NPs were properly formulated and conjugated for effective drug delivery, the size, zeta potential, and chemical properties of the NPs were evaluated after formulation. Dynamic light scattering (DLS) was utilized to measure the size and zeta potential of the PTX-S2.2-NPs and HNK-S2.2-NPs (Figure 2A). The results show that the sizes of the PTX and HNK NPs after conjugation were 83.4 nm (PDI 0.513) and 163 nm (PDI 0.179), respectively. We found that the zeta potential of the PTX-S2.2-NPs and HNK-S2.2-NPs are −9.74 and −7.16, respectively.

#### 2.2.2. Fourier-Transform Infrared Spectroscopy (FTIR)

FTIR spectroscopy was performed to validate the appropriate conjugations of the PTX-S2.2 PBM NPs and HNK-S2.2 PBM NPs. PEGylation has been shown to lead to longer circulation time when compared to NPs without PEGylation; it also reduces the accumulation in the reticuloendothelial system (RES) and prevents opsonization [26]. On the other hand, PEG copolymer PCL is biodegradable and biocompatible, allows for controlled drug delivery and reduced toxicity, and has a slower degradation rate [27]. The delivery of the NPs can be further enhanced beyond the EPR effect via the functionalization of nanocarriers with ligands such as aptamers. Verifying proper conjugation of the PBM NPs is important to validate the potential efficacy of the NPs. To do so, FTIR was performed to obtain the spectrum of HNK, PTX, unconjugated HNK NPs coated with PCL/PEG, unconjugated PTX NPs coated with PCL/PEG, and aptamer-conjugated HNK and PTX NPs.

When analyzing the HNK, unconjugated HNK, and conjugated HNK NPs’ spectra, peaks at 1496, 1498, and 1442 cm^−1^ were due to aromatic C=C or CH_2_ bending, indicating the presence of the aromatic ring from HNK (Figure 2B). Peaks at 3296, 3286, 3315 cm^−1^ in the HNK, unconjugated HNK-NPs, and HNK-S2.2-NPs, respectively, confirm the presence of O–H/N–H stretch. These results indicate the presence of HNK and PEG. The shift seen in the HNK-S2.2-NPs’ spectra suggests the presence of the aptamer [28]. Peaks 825, 825, and 795 cm^−1^ indicate the presence of C–H bending or an aromatic ring out-of-plane. These findings demonstrate that the HNK fingerprint was retained in all. The aptamer presence was further suggested by a peak at 1654 cm^−1^, which was only observed in the conjugated NPs’ spectra, indicating a C=O stretch in the Amide I region [29]. Peaks of Amide II were also observed at 1220, 1218, and 1215 cm^−1^, respectively. A stronger peak was observed in the HNK-S2.2-NPs’ spectra, indicating the presence of the aptamer. PCL and PEG coating were confirmed with peaks at 2900 and 2924 cm^−1^ in unconjugated and conjugated HNK NPs, respectively [30]. These results imply aliphatic C–H stretching, confirming the presence of the polymer backbone in the PCL/PEG chain. An ether C–O–C stretch was also observed at 1005, 995, 1013 cm^−1^ in the spectra of HNK, unconjugated HNK, and conjugated HNK. These findings are indicative of the presence of PEG.

Similarly, PTX, unconjugated PTX, and conjugated PTX NPs’ spectra analysis revealed peaks at 3481, 3485, and 3530 cm^−1^ due to O–H/N–H stretching (Figure 2C). The aptamer and the O–H of PTX contribute to the N–H. The presence of PTX was further confirmed by peaks at 1146, 1155, and 1150 cm^−1^ in PTX, unconjugated PTX, and conjugated PTX NPs’ spectra, respectively, due to the C–O–C stretch from paclitaxel’s cyclic ether and PEG in formulations. The presence of peaks 1750 and 2900 cm^−1^ in both unconjugated and conjugated PTX spectra is due to C=O contributed by the ester carbonyl from PCL and C–H aliphatic stretching, respectively. These results confirm the presence of the polymer backbone and PCL/PEG chains. A peak at 1735 cm^−1^ due to C=O, contributed by the Paclitaxel ester, was observed in the PTX spectra but is not found in the unconjugated and conjugated PTX NPs. The disappearance of this peak suggests the bonding/entrapment of PTX within the NPs. Peaks 1647, 1640, and 1650 cm^−1^ seen in PTX, unconjugated PTX, and conjugated PTX NPs’ spectra are due to the Amide I C=O in the peptide bond. The shift observed in the conjugated PTX NPs spectrum confirms the aptamer conjugation via amide linkage. Peaks 1450 and 1440 cm^−1^, in PTX and conjugated PTX NPs’ spectra, respectively, are due to C=C aromatic/CH_2_ bending, indicating the paclitaxel and/or aptamer aromatic structure. The presence of the aptamer was further validated by peaks at 1244, 1240, and 1211 cm^−1^, corresponding to the amide bend of the aptamer C–N stretch. The peak of the aptamer is more pronounced in conjugation, confirming the aptamer presence. Overall, the findings from FTIR spectra analysis suggest that conjugated PTX and HNK NPs were synthesized correctly, supporting their potential efficacy.

#### 2.2.3. High-Performance Liquid Chromatography (HPLC) Analysis

The encapsulation efficiency (EE%) of paclitaxel (PTX) and honokiol (HNK) encapsulated within planetary ball-milled (PBM) nanoparticles was systematically quantified using high-performance liquid chromatography (HPLC). For the PTX-PBM NPs formulation, the encapsulation efficiency was calculated to be 77%, reflecting a high level of drug retention during nanoparticle fabrication (Figure 2D,E).

In the case of HNK-PBM NPs, an even higher encapsulation efficiency of 84.08% was achieved (Figure 2F,G). This result signifies exceptional entrapment performance and suggests a strong compatibility of honokiol with the PBM matrix. The superior loading observed for HNK relative to PTX may be attributed to differences in drug–polymer interactions, hydrophobicity, or molecular structure that favor higher incorporation during encapsulation.

These findings confirm efficient encapsulation of the PBM-based nanoparticle system for the efficient encapsulation of hydrophobic anticancer agents. The high EE% achieved for PTX and HNK are critical parameters for effective drug delivery, prolonged release kinetics, and reduced systemic toxicity. These characteristics underscore the potential of PBM nanoparticles as a promising platform for targeted and sustained drug delivery in cancer therapeutics.

### 2.3. Molecular Docking Studies

**Figure 3:**
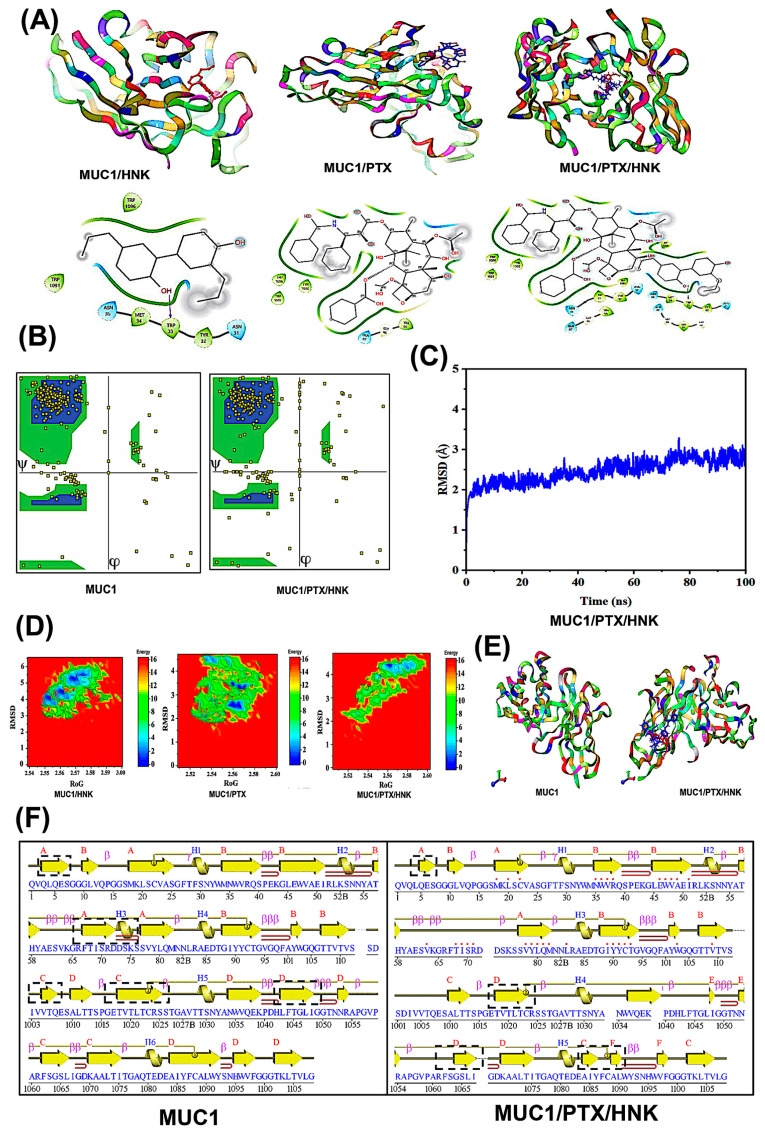
Structural and dynamic characterization of MUC1 in complex with HNK, PTX, and PTX–HNK via molecular docking and molecular dynamics simulations: (**A**) Molecular docking of ligands with MUC1. Ribbon representations of the MUC1–HNK, MUC1–PTX, and MUC1–PTX–HNK complexes showing the binding orientation of ligands (upper row), and 2D interaction diagrams (lower row) highlighting key interactions such as hydrogen bonding and hydrophobic contacts. (**B**) Ramachandran plot analysis. Comparison of the stereochemical quality of native MUC1 (left) and the MUC1–PTX–HNK ternary complex (right). Yellow dots represent φ–ψ dihedral angles; green, blue, and white regions denote allowed, favored, and disallowed regions, respectively. (**C**) Root Mean Square Deviation (RMSD). Time evolution of backbone RMSD over 100 ns for the MUC1–PTX–HNK complex, indicating structural equilibration and overall stability throughout the simulation period. (**D**) Free Energy Landscapes (FEL). Two-dimensional potential energy surfaces projected along the radius of gyration (RoG) and RMSD for MUC1–HNK (left), MUC1–PTX (middle), and MUC1–PTX–HNK (right) systems. The color scale indicates energy, ranging from low (blue) to high (red), with distinct minima revealing conformational stability and thermodynamic favorability. (**E**) 3D structural superposition. Overlay of native MUC1 (left) and MUC1–PTX–HNK complex (right), showing localized conformational changes upon ligand binding, particularly in surface loops and binding-site-adjacent helices. (**F**) Secondary structure map. Comparison of residue-level secondary structure assignments for native MUC1 (left) and the ternary MUC1–PTX–HNK complex (right). Structural elements include β-sheets, α-helices, β-turns, and γ-turns, indicating ligand-induced shifts toward increased structural ordering.

Docking studies revealed that honokiol (HNK) and paclitaxel (PTX) exhibit favorable binding to the tandem-repeat domain of MUC1 (Figure 3A). The MUC1/HNK complex showed a binding affinity of –8.1 kcal/mol, while MUC1/PTX exhibited a slightly stronger interaction with a ΔGbinding of –8.7 kcal/mol, as predicted by AutoDock Vina v1.2. These findings suggest the formation of energetically favorable non-covalent interactions, primarily stabilized by hydrogen bonds, π–π stacking, and hydrophobic contacts within the MUC1 binding cleft. These interactions are consistent with previously reported binding characteristics of MUC1-targeting small molecules, particularly those that exploit the proline-rich and hydrophobic surface topology of the tandem-repeat region.

The ternary complex (MUC1/PTX/HNK) maintained dual occupancy within the receptor’s binding cleft, yielding a combined binding energy of –9.4 kcal/mol, suggesting favorable interactions. The docking poses demonstrated that both ligands could bind simultaneously without significant steric clashes, supporting the feasibility of co-binding. The simultaneous accommodation of both ligands without steric hindrance suggests that their binding sites are either non-overlapping or minimally overlapping and may, in fact, stabilize one another through cooperative effects. This supports a potential mechanism where PTX binds deeply within the core binding groove while HNK anchors more peripherally, possibly reinforcing structural rigidity or altering local conformational dynamics.

Such co-binding behavior may have important therapeutic implications. This implies that a combinatorial drug strategy targeting MUC1 with PTX and HNK could exert complementary effects, with PTX contributing to microtubule disruption. At the same time, HNK modulates signaling or epigenetic factors, thus enhancing overall anticancer efficacy. The ability of both ligands to simultaneously engage the MUC1 tandem-repeat domain without inducing unfavorable steric or energetic penalties supports the rational design of multi-ligand nanocarrier systems for improved delivery and specificity in MUC1-overexpressing cancers.

### 2.4. Root Mean Square Deviation (RMSD)

RMSD analysis was performed over a 100-ns simulation period to assess the structural stability of the MUC1–ligand complexes. The MUC1/PTX complex exhibited the highest average RMSD value of 4.21 Å, followed by MUC1/HNK with an RMSD of 3.94 Å, indicating moderate structural fluctuations upon ligand binding (Figure 3C). In contrast, the ternary complex MUC1/PTX/HNK demonstrated the lowest RMSD value of 2.56 Å, suggesting a more stabilized conformation. All systems showed an initial equilibration phase within the first 10–20 ns, followed by a stable plateau for the remainder of the simulation.

### 2.5. Ramachandran Plot

The Ramachandran plot analysis revealed that the native MUC1 structure exhibited 85.4% of residues in the most favored regions, 8.5% in additionally allowed regions, 5.1% in generously allowed regions, and 1.0% in the disallowed areas, with a G-factor of −0.15 (Figure 3B). The modest proportion of residues in disallowed and generously allowed regions in the native MUC1 structure suggests inherent conformational flexibility or areas prone to structural strain.

For the MUC1/PTX complex, 85.4% of residues were in the most favored regions, 8.5% in additionally allowed regions, 5.1% in generously allowed regions, and 1.0% in the disallowed areas (G-factor: −0.15). In contrast, in the MUC1/HNK complex, there was a noticeable shift toward more favorable backbone dihedral angles, evidenced by an increase in the most favored regions to 88.6%, 10.7% in additionally allowed regions, 0.2% in generously allowed regions, and a reduction in disallowed residues to 0.4% (G-factor: −0.12). The progressive improvement in the Ramachandran statistics across the MUC1 complexes indicates a stabilizing effect upon ligand binding.

The MUC1/PTX/HNK complex observed the most significant enhancement. The MUC1/PTX/HNK ternary complex demonstrated the highest stereochemical quality, with 91.2% residues in the most favored regions, 7.8% in additionally allowed regions, only 0.7% in generously allowed regions, and a minimal 0.3% in the disallowed areas, supported by a positive G-factor of +0.21. The positive G-factor further reflects improved overall stereochemical quality and reduced energetic strain in the protein backbone. This suggests that dual ligand binding induces conformational refinement and structural tightening, particularly around the tandem-repeat domain, which likely enhances stability. Minimizing energetically unfavorable backbone conformations in the ternary complex supports the cooperative and stabilizing interaction hypothesis between PTX, HNK, and MUC1.

### 2.6. Free Energy Landscape (FEL)

FEL of the MUC1/HNK complex, projected along the RMSD (2.5–6.0 Å) and radius of gyration (RoG ~2.54–2.90 nm), revealed one major low-energy basin at ~3.8 Å RMSD and ~2.57 nm RoG with multiple shallow minima, suggesting diverse conformational states (Figure 3D). The minimum free energy in this system reached ~0.5 kcal/mol, while high-energy regions exceeded 16 kcal/mol.

For the MUC1/PTX complex, the FEL extended over an RMSD range of 1.5–4.5 Å and RoG of ~2.52–2.60 nm, with the primary energy well located around 2.6 Å RMSD and 2.56 nm RoG, indicating a compact and moderately fluctuating conformational state. The energy minima reached ~0.2–0.3 kcal/mol, surrounded by low-energy valleys, indicating semi-stable intermediate conformations.

The MUC1/PTX/HNK ternary complex exhibited a tighter and broader global minimum with reduced dispersion across conformational space. It had a well-defined basin around 2.4–3.2 Å RMSD and 2.55–2.58 nm RoG, with the lowest energy reaching nearly 0.0–0.1 kcal/mol, indicating exceptional thermodynamic stability. The energy landscape here was smoother, with fewer local basins, suggesting a well-optimized conformational ensemble. Overall, these FEL profiles validate the ternary complex’s improved energetic and structural stability, reinforcing the hypothesis that dual-targeting of MUC1 by PTX and HNK promotes structural consolidation and could offer enhanced therapeutic efficacy in MUC1-driven cancers.

### 2.7. Free Binding Energy

MM-PBSA calculations were performed to estimate the free binding energy (ΔG) and its energetic components for MUC1 in complex with Honokiol (HNK), Paclitaxel (PTX), and the dual-ligand system (MUC1/PTX/HNK). The total binding free energies reveal that all three complexes are thermodynamically stable, as indicated by highly negative ΔG values −66.05 ± 0.07 kcal/mol for MUC1/HNK, −60.17 ± 0.42 kcal/mol for MUC1/PTX, and significantly more favorable at −80.72 ± 0.21 kcal/mol for the MUC1/PTX/HNK ternary complex. This enhanced stability likely results from synergistic effects of dual-ligand binding, where PTX and HNK engage complementary binding sites on MUC1, thereby maximizing intermolecular interactions.

In terms of energy components, the electrostatic interaction energy (ΔEelectrostatic) was most negative in the ternary system (–53.78 ± 0.85 kcal/mol) compared to –42.85 ± 0.59 kcal/mol for PTX and –38.91 ± 0.56 kcal/mol for HNK, indicating stronger Coulombic stabilization when both ligands are present. Similarly, van der Waals interactions (ΔEvdw) contributed significantly across all systems, with the most favorable value for the ternary complex (–48.82 ± 0.15 kcal/mol), followed by HNK (–45.27 ± 0.51 kcal/mol) and PTX (–41.56 ± 0.82 kcal/mol). The markedly more negative electrostatic and van der Waals energies in the ternary complex suggest dense, cooperative molecular packing and enhanced contact with the protein surface.

Solvation energies showed opposing effects: the polar solvation term (ΔGGB) was highest in the ternary complex (+17.8 ± 0.01 kcal/mol), acting unfavorably, while nonpolar solvation (ΔGSA) was consistently stabilizing, with values ranging from –11.24 to –14.27 kcal/mol. Enthalpy contributions (ΔH) were similar across all systems (~18 kcal/mol), whereas the entropic penalty (–TΔS) was lowest in the ternary complex (16.04 ± 0.10 kcal/mol) compared to 21.66 ± 0.39 kcal/mol for PTX and 19.79 ± 0.22 kcal/mol for HNK. Although the polar solvation penalty is highest in the ternary system, it is outweighed by the strong direct interactions and minimized entropic cost. The lower entropy penalty (–TΔS) in the dual-ligand system implies reduced conformational freedom upon binding, possibly due to induced structural ordering of MUC1.

### 2.8. 3D Structural Superposition and Secondary Structure Map

The comparative analysis of the 3D structures of native MUC1 and the MUC1/PTX/HNK ternary complex revealed distinct structural modulations upon ligand binding (Figure 3E). Superposition of the two structures demonstrated localized conformational shifts primarily in loop regions adjacent to the ligand-binding cleft, particularly in the β-turns and surface-exposed helices. These alterations were most pronounced in areas surrounding the bound PTX (red) and HNK (blue), indicating that ligand anchoring influenced side-chain reorientation and minor backbone adjustments. Notably, the global fold of MUC1, including the central β-sheet scaffold and overall tertiary architecture, remained unchanged, suggesting a structurally conserved framework during complex formation. Furthermore, the close packing of PTX and HNK within the binding site reinforces the hypothesis of cooperative ligand binding, where one ligand may stabilize the binding conformation of the other. This spatial synergy contributes to enhanced interaction energy and reduced entropy, consistent with earlier observations from free energy and RMSD analyses.

The secondary structure assignment of native MUC1 and its PTX/HNK-bound ternary complex reveals distinct structural rearrangements upon ligand binding (Figure 3F). The native MUC1 structure features 4 β-sheets, 8 β-hairpins, 3 β-bulges, 22 β-strands, 6 α-helices, 22 β-turns, 3 γ-turns, and 2 disulfide bonds. In contrast, the MUC1/PTX/HNK complex exhibits 6 β-sheets, a slight reduction to 6 β-hairpins and 1 β-bulge, 20 β-strands, 5 α-helices, but a slight increase in β-turns (24) and maintenance of 2 disulfide bonds. The formation of additional β-sheets and β-turns in the ternary complex suggests a stabilizing effect on loop regions, where flexible coil or disordered segments transition into more ordered structural elements upon ligand binding. The presence of fewer β-bulges and hairpins in the ternary complex may reflect smoother topology and packing, consistent with the induced-fit model and the formation of a more compact, energetically favorable tertiary structure, as also supported by the Free Energy Landscape and MM-PBSA analysis. Such structural evidence strengthens the rationale for using dual-drug targeting approaches.

### 2.9. Cytotoxic Effect of Aptamer Conjugated PTX and HNK NPs on Breast Cancer Cells

**Figure 4:**
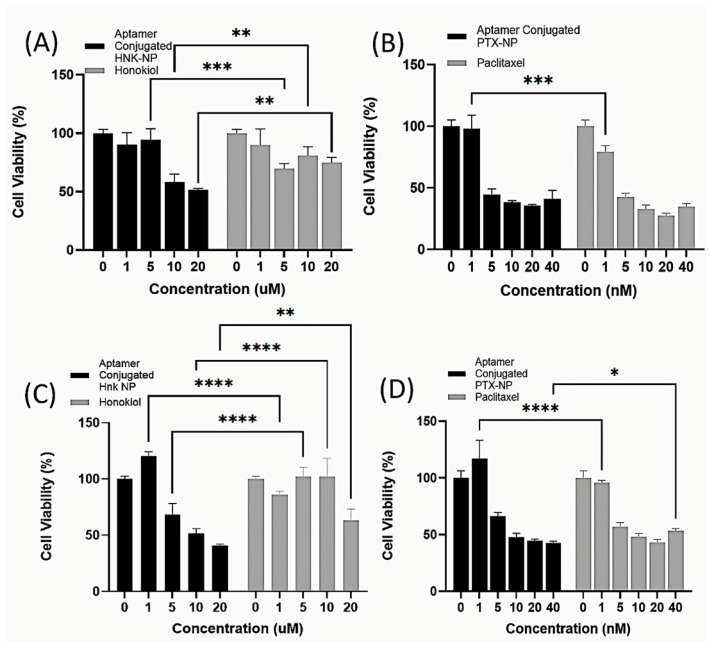
Cytotoxic effect of honokiol (HNK) and aptamer conjugated HNK nanoparticle in HCC70 cells (**A**), paclitaxel (PTX) and aptamer conjugated HNK nanoparticle in HCC70 cells (**B**), PTX and aptamer conjugated PTX nanoparticle in MDA MB 468 cells (**C**), and PTX and aptamer conjugated PTX nanoparticle in MDA MB 468 cells (**D**). The HCC 70 and MDA MB 468 cells were treated with HNK/HNK-NPs at concentrations 0, 1, 5, 10, 20 μM and PTX/PTX NPs at concentrations 0, 1, 5, 10, 20 nM for 72 h. Cell viability is presented as mean± SD (*n* = 3); signifying * *p* < 0.05, ** *p* < 0.005, *** *p* < 0.0005, **** *p* < 0.00005.

HCC70 and MDA-MB-468 cell lines were treated with HNK-S2.2-NPs/free HNK at concentrations of 0, 1, 5, 10, 20 μM and PTX-S2.2-NPs/free PTX at concentrations of 0, 1, 5, 10, 20, 40 nM for 72 h. Subsequently, an MTT assay was performed to assess the anticancer properties of the conjugated NPs. Compared to the controls, both HNK-S2.2-NPs and PTX-S2.2-NPs reduced the proliferation of HCC 70 and MDA-MB-468 cells in a time- and dose-dependent manner. At 72 h, HNK-S2.2-NPs showed a significantly greater decrease in cell viability in HCC70 cells when compared to free HNK at 10 μM (*p* < 0.005) and 20 μM (*p* < 0.005) concentrations (Figure 4A). Similarly, when compared to free HNK, a significant decrease in MDA-MB-468 cell viability was observed at 72 h at concentrations 1 (*p* < 0.0005), 5 (*p* < 0.0005), 10 (*p* < 0.0005), and 20 μM (*p* < 0.005) (Figure 4C). However, after 72 h, when compared to free PTX, a significant decrease in HCC 70 cell viability was not observed after PTX-S2.2-NP treatment (Figure 4B). In contrast, a substantial reduction in cell viability of MDA-MB-468 cells after 72 h of PTX-S2.2-NP treatment was observed at 40 nM (*p* < 0.05) when compared to free PTX (Figure 4D). When PTX and HNK S2.2. NPs are used in combination synergism is observed (Supplementary Figure S1). The following IC50 values were established at 72 h for HCC70: 5.822 (PTX), 6.94 nM (PTX-S2.2-NPs), 48.21 μM (HNK), and 15.41 μM (HNK-S2.2-NPs). The following IC50 values were established for MDA-MB-468 at 72 h: 10.18 nM (PTX), 11.25 nM (PTX-S2.2-NPs), 49.73 μM (HNK), and 13.02 μM (HNK-S2.2-NPs). The presented results demonstrate the efficacy of the PTX-S2.2-NPs and HNK-S2.2-NPs when compared to their free drug counterparts.

### 2.10. Live/Dead Cell Viability Assay

The use of fluorescence imaging to determine cell viability by exploiting the loss of the membrane permeability barrier in nonviable cells is considered a more accurate and reliable method for assessing cell viability [31]. In the present study, NucBlue^®^ Live reagent was used to stain the nuclei of the live cells and was detected using a DAPI filter, while the NucGreen^®^ Dead reagent stained the nuclei of the dead cells that had a compromised plasma membrane and was detected with the FITC filter.

**Figure 5:**
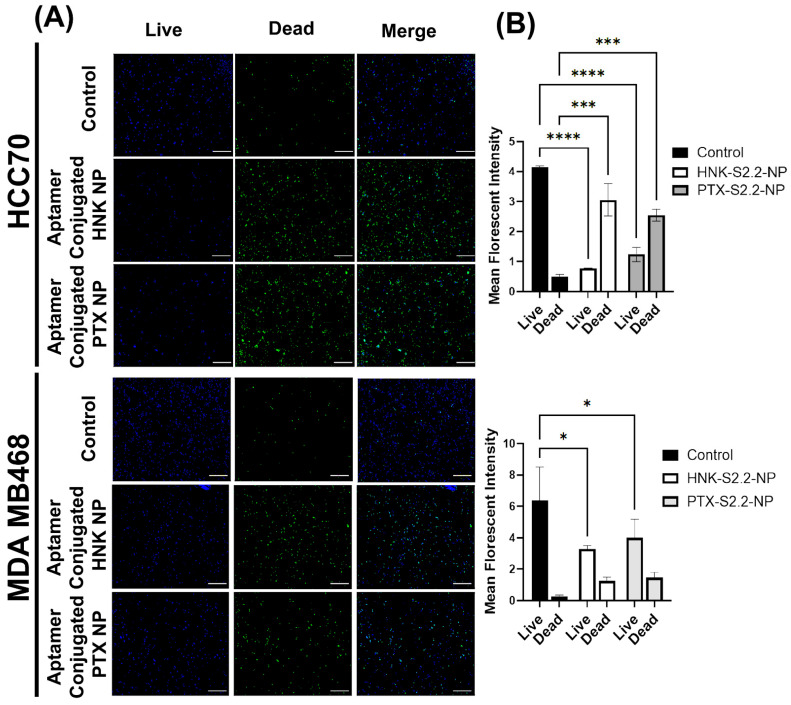
Live/dead cell assay. (**A**) A live/dead cell viability assay of HCC70 and MDA-MB-468 cells was performed using the Blue/Green ReadyProbes^®^ cell viability imaging kit. NucBlue^®^ Live reagent was used to stain the nuclei of the live cells and was detected using a DAPI filter, while the NucGreen^®^ Dead reagent stained the nuclei of the dead cells that had a compromised plasma membrane and was detected with the FITC filter (IX73 Inverted Microscope, Olympus Corporation of the Americas, Center Valley, PA, USA). (**B**) Mean Fluorescence intensities of live and dead images were quantified using ImageJ (version 1.54g). Scale bar represents 50 μm. Fluorescent intensity is presented as mean ± SD (*n* = 3); signifying * *p* < 0.05, *** *p* < 0.0005, **** *p* < 0.00005.

Prior to imaging, for 72 h, HCC70 treatment wells were treated with predetermined IC50 values of 6.94 nM (PTX-S2.2-NPs) and 15.41 μM (HNK-S2.2-NPs), while MDA-MB-468 treatment wells were treated with values of 11.25 nM (PTX-S2.2-NPs) and 13.02 μM (HNK-S2.2-NPs). Compared to the untreated HCC70 and MDA-MB-468 cells, there was an increase in green fluorescence and a decrease in blue fluorescence after treatment with PTX-S2.2-NPs and HNK-S2.2-NPs. After treatment, a significant change in mean fluorescent intensity was observed in both live and dead HCC70 cells with mean intensities of 0.778 ± 0.006 (*p* < 0.00005) and 3.053 ± 0.539 (*p* < 0.0005) after HNK-S2.2-NP treatment, and 1.245 ± 0.2242 (*p* < 0.00005) and 2.551 ± 0.2 (*p* < 0.0005) after PTX-S2.2-NP treatment (Figure 5). In contrast, MDA-MB-468 cells significantly decreased live cells after PTX-S2.2-NPs and HNK-S2.2-NP treatment when compared to the control mean fluorescent intensity of 6.370 ± 2.133. The mean fluorescent intensities for live cells were 3.284 ± 0.221 (*p* < 0.05) and 4.002 ± 1.179 (*p* < 0.05) after HNK-S2.2-NPs and PTX-S2.2-NP treatment, respectively.

## 3. Discussion

PTX is a widely used neo-adjunctive and adjunctive BrCa treatment; however, though known to be efficacious, when PTX is used as a monotherapy, there are disadvantages, such as reduced tolerability due to drug nonspecificity and drug resistance, often due to drug efflux [32,33]. As a result, targeted combination therapies have become essential in treating BrCa. HNK is a natural, nontoxic compound derived from the magnolia tree that is known to work synergistically with PTX and can reverse and overcome drug resistance in cancer via the downregulation of key drug resistance-related pathways and inducing apoptosis [34].

Treatments utilizing free drugs often result in off-target effects leading to an increase in toxicity and adverse health outcomes. However, carrier encapsulation could lead to an increased specificity towards cancerous cells, helping to ensure the drug’s efficacy and improve bioavailability. In this study, we utilized PBM NPs. This novel drug transporter system used a receptor-based targeted delivery approach by coating the NPs with MUC1 aptamer (S2.2) and a PCL-PEG copolymer. Functionalized NPs conjugated to target overexpressed receptors, such as folate receptor, have been shown to be very valuable for cancer treatment and theranostic approaches to BrCa management [35]. Similarly, MUC1 has emerged as a viable drug target for many cancers due to its oncogenic nature and increased expression in most cancer types, including BrCa [36]. In this study, MUC1 expression in BrCa was confirmed through TMA slide analysis, which exhibited, when compared to normal breast tissue, a significant increase in MUC1 expression, with Stage 4 BrCa having the highest expression. Through in silico methods, our findings suggest that PTX and HNK, when used in combination, exhibit increased binding affinity and bind to the same pocket when targeting MUC1, reinforcing the rationale for using dual-drug therapies in treating BrCa.

After PTX-S2.2 and HNK.S2.2 synthesis via NHS coupling, the NPs were characterized using DLS and FTIR. The findings revealed that the zeta potential of the PTX-S2.2-NPs and HNK-S2.2-NPs is −9.74 and −7.16, respectively. NPs with a slightly negative or neutral zeta potential have prolonged circulation in the body because they are less susceptible to opsonization and clearance by the reticuloendothelial system. It is important to note that there is a potential for aggregation the closer the zeta potential is to zero; however, PEGylation has been shown to stabilize the NPs, preventing aggregation via steric hindrance [37,38,39,40]. Similarly, the size of HNKS2.2 (163 nm) and PTXS2.2 NPs (83.4 nm) fall within or close to ~100–200 nm which is the size range in which NPs can passively accumulate in tumors via the enhanced permeability and retention effect (EPR), which can potentially result in lower toxicity in normal tissues and higher therapeutic effects in tumors [41,42,43]. FTIR peak analysis suggested that the NPs were properly encapsulated and conjugated. Further characterization is necessary to validate these findings.

Furthermore, we demonstrated through MTT and live-dead cell assays that PTX and HNK, when encapsulated in S2.2-PCL-PEG-coated PBM NPs, caused significant cell death compared to untreated cells. However, compared to MDA MB 468, a more substantial effect on cell viability was observed in HCC 70 cells, which are known to have high MUC1 expression (Supplementary Figure S2). Also, we discovered that the unconjugated NPs were equally cytotoxic as the free drugs. This may indicate that the level of MUC1 expression could have potentially impacted the effects of the aptamer-conjugated drug treatment. These findings demonstrate that the treatments may be more effective in tumors with higher MUC1 expression. Overall, our study further validates the efficacy of the aptamer-conjugated NPs for BrCa treatment.

## 4. Materials and Methods

### 4.1. Materials

5′amino-modified MUC1 DNA Aptamer S2.2 with sequence of 5′Amine-C12-GCAGTTGATCCTTTGGATACCCTGG-3″ was synthesized by Aptagen LLC (Jacobus, PA, USA). Polyethylene glycol 8000 was purchased from Research Organics (Cleveland, OH, USA). Polycaprolactone, N, N″ disuccinimidyl carbonate, and 4-dimethylamino pyridine solution were purchased from Sigma-Aldrich (St. Louis, MO, USA). Honokiol was purchased from MedChemExpress (Monmouth Junction, NJ, USA). RPMI 1640 medium, L-15 medium, fetal bovine serum (FBS), 0.25% trypsin, paclitaxel, starch, 1,4-dioxane, N-hydroxysuccinimide, and acetone were purchased from Fisher Scientific (Pittsburgh, PA, USA).

### 4.2. Breast Cancer Cell Lines

The hypothesis was tested using HCC 70 and MDA-MB-468 cancer cell lines. The BrCa cell lines were procured from the American Tissue Culture Collection (ATCC, Manassas, VA, USA). HCC 70 cells were grown and cultured in RPMI-1640 media, supplemented with 10% fetal bovine serum (FBS), 1% nonessential amino acids, 1% HEPES, 2 mM L-glutamine, and 1% penicillin/streptomycin antibiotic solution (Fisher Scientific, Pittsburgh, PA, USA). However, MDA-MB-468 was cultured in L-15 media, supplemented with 10% fetal bovine serum (FBS), 1% HEPES, 1% nonessential amino acids, and 1% penicillin/streptomycin antibiotic solution (Fisher Scientific, Pittsburgh, PA, USA). Cell lines were then maintained in an incubator at 37 °C and with 5% CO_2_.

### 4.3. Immunohistochemistry

To investigate the expression of MUC1 in human tissue, the human tissue microarray (TMA) containing 72 cases from stages I to IV was obtained from the US BIOMAX, Inc. (Derwood, MD, USA). The TMA staining was performed following the standard protocol [7]. Paraffin-embedded tissue sections were deparaffinized in xylene and rehydrated through a graded alcohol series. They were washed with deionized water and phosphate-buffered saline (PBS) for 5 min, followed by antigen retrieval to enhance immunogenicity and epitope availability. Subsequently, sections were washed with PBS-T (T: Twin 20), blocked with H_2_O_2_, stained with MUC1 primary antibody (1:350) (overnight) (Abcam Inc., Cambridge, MA USA), followed by secondary (R&D Systems, Pittsburgh, PA, USA). Finally, the tissue sections were developed using the 3,3′-Diaminobenzidine (DAB) coloring agent, counterstained with hematoxylin, dehydrated, and mounted. The images were captured using the AperioScanScope scanning system (Aperio Technologies, Deer Park, IL, USA), and data were analyzed using MIKAIA software (version 2.4) (Fraunhofer IIS, Erlangen, Germany).

### 4.4. Molecular Docking and Dynamics Simulation Studies

The molecular docking and dynamics simulation studies were carried out to investigate the binding interactions between MUC1 and the ligands Honokiol (HNK) and Paclitaxel (PTX), both individually and in combination. The molecular docking and dynamics simulations were performed as exploratory analyses to evaluate whether PTX and HNK could theoretically be accommodated within the MUC1 binding cleft, thereby supporting the conceptual basis for co-delivery in a MUC1-targeted nanoparticle system. The X-ray crystal structure of the tandem-repeat domain of human MUC1 (PDB ID: 6BSB; 1.95 Å resolution) was retrieved from the Protein Data Bank. Honokiol (PubChem CID: 82147) and Paclitaxel (CID: 36314) were obtained in SDF format and converted to MOL2 files using Open Babel 3.2. Rigid-receptor, flexible-ligand docking was performed using AutoDock Vina 1.2 for three systems: MUC1/HNK, MUC1/PTX, and MUC1/PTX/HNK. A grid box of 30 × 30 × 30 Å was centered on the carbohydrate-binding cleft of MUC1 to encompass key residues involved in ligand interaction. Docking exhaustiveness was set to 24, and the top-ranked pose based on binding affinity (ΔG_binding_) was selected for each complex. For the ternary complex, the MUC1/PTX structure was used as the receptor for subsequent docking with HNK.

The best-scoring complexes from docking were subjected to molecular dynamics (MD) simulations using GROMACS v2023.4. The CHARMM36 m force field was applied for the protein, and ligand topologies were generated via CHARMM-GUI using the CGenFF protocol. Each complex was solvated in a cubic TIP3P water box with a 10 Å buffer and neutralized with Na⁺ and Cl^−^ ions. Energy minimization was performed using the steepest-descent algorithm until the maximum force was below 1000 kJ mol^−1^ nm^−1^. Equilibration was conducted in two phases: a 100 ps NVT ensemble at 310 K using the V-rescale thermostat (τ = 0.1 ps), followed by a 100 ps NPT ensemble at 1 bar using the Parrinello–Rahman barostat (τ = 2.0 ps), with all heavy atoms restrained by a force constant of 1000 kJ mol^−1^ nm^−2^. Production MD was run for 100 ns per system with a 2 fs time step, periodic boundary conditions, and LINCS constraints on hydrogen bonds. Long-range electrostatic interactions were handled via the particle mesh Ewald (PME) method with a cutoff of 1.2 nm. Trajectories were recorded every 10 ps for subsequent analysis.

Trajectory analysis was conducted using GROMACS and MDAnalysis. Structural stability was assessed by calculating the backbone root-mean-square deviation (RMSD) and Ramachandran plot. Protein–ligand interactions were characterized by hydrogen bonding and hydrophobic contacts throughout the simulations. Binding free energies were estimated using the molecular mechanics Poisson–Boltzmann surface area (MM-PBSA) approach implemented via gmx MMPBSA, using 50 snapshots extracted at 2 ns intervals to evaluate the relative stability of the complexes.

### 4.5. Planetary Ball Milling (PBM) NPs Formulation

This method requires the use of a milling jar and heat-absorbing zirconium oxide planetary milling balls. The jar rotates around an axis, causing the milling balls within the jar to rotate, thereby milling the macroparticles containing 4% starch and the drugs. The size of the particles is controlled by adjusting the centrifugal force through varying the revolutions/sec (Ω), jar velocity, the size and number of zirconium oxide balls, duration, and the number of cycles, which all influence the particle size [9].

### 4.6. Preparation of S2.2 Aptamer-Conjugated PEG via NHS Activation and Nanoparticle Functionalization

**Scheme 1:**
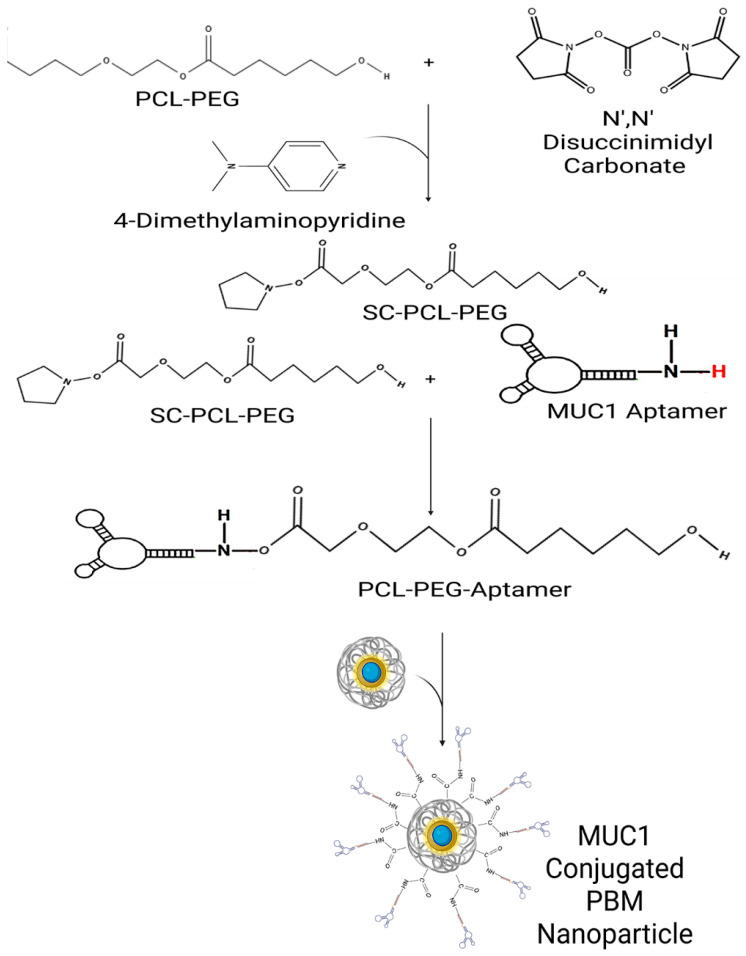
Schematic of the synthesis of MUC1 aptamer-conjugated PBM nanoparticles via NHS crosslinking. Created in BioRender. Kinnel, B. (2025) https://BioRender.com/ffimqs0.

For the PEG activation, 5 g of PEG 8000 was dissolved in 25 mL of dioxane by heating in a water bath until completely dissolved. 6 mM of N, N″ Disuccinimidyl carbonate (DSC) and 6 mM of 4-diethylamino pyridine were dissolved in 10 mL of acetone via stirring. The DSC and pyridine solution were added to the PEG solution slowly and then reacted overnight. Diethyl ether is added to the solution to precipitate out the SC-PEG. The precipitant was redissolved in acetone and then reprecipitated using diethyl ether. The precipitant was dissolved and precipitated at least once more before being left to dry to become a powder. For PCL activation, 2 g PCL was dissolved in 6 mL of dioxane by heating in a water bath until completely dissolved. Next, DSC and diethylamino pyridine were dissolved in acetone and added to the PCL solution while stirring. The solution was stirred overnight to react, and then it was filtered. Diethyl ether was added to precipitate the product, and then it was left to dry. The activated PCL and PEG were then dissolved in methylene chloride, and then the S2.2 aptamer stock solution (25 μM) was added dropwise and stirred for 30 min. The NPs were then dissolved in methylene chloride and added to the PCL/PEG/Aptamer solution (Scheme 1). This mixture was left to react for at least 3 h. The product was then lyophilized overnight.

### 4.7. Characterization of Breast Cancer Cell-Specific PBM Nanoparticles

#### 4.7.1. Size and Zeta Potential

The size and zeta potential of PTX-S2.2-NPs and HNK-S2.2-NPs were measured using a Malvern Zetasizer ZS instrument (Malvern Panalytical, Westborough, MA, USA) at a concentration of 0.1 mg/mL (5% mass, assuming a density of 1 g/cm^3^) of nanoparticles.

#### 4.7.2. High-Performance Liquid Chromatography (HPLC)

Quantitative analysis of PTX and HNK encapsulated in PBM nanoparticles was performed using a high-performance liquid chromatography (HPLC) system equipped with a quaternary pump, autosampler, and UV–visible detector (Agilent 1260 Infinity Quaternary system, Agilent, Santa Clara, CA, USA). Chromatographic separation was achieved using a C18 reversed-phase column (250 mm × 4.6 mm, 5 µm particle size) maintained at ambient temperature.

For PTX analysis, the mobile phase consisted of methanol and water (80:20, *v*/*v*) with a flow rate of 1.0 mL/min. Detection was performed at a wavelength of 230 nm. The injection volume was 50 µL, and the total runtime was 15 min per sample.

HNK analysis was conducted using a mobile phase of methanol and water (80:20, *v*/*v*) under isocratic conditions with a flow rate of 1.0 mL/min. UV detection was performed at 290 nm. The injection volume was 50 µL, with a total runtime of 10 min.

Standard calibration curves for both PTX and HNK were prepared by dissolving accurately weighed amounts of the pure drugs in DMSO and further diluting with the appropriate mobile phase to obtain a series of standard solutions at known concentrations (ranging from 1.0 to 100 µg/mL for PTX and 1.5 to 24 mM for HNK). Each concentration was injected in triplicate, and the resulting chromatograms were used to calculate the area under the curve (AUC). Linear regression was applied to the plotted concentration vs. AUC data to generate the standard curves, which were used for subsequent quantification of drug content in nanoparticle samples.

The encapsulated drug concentration was calculated by subtracting the free drug detected in the supernatant from the total amount initially used in the formulation. Encapsulation efficiency (EE%) was calculated using the following formula:

$$EE\left(\%\right)=\frac{Total\;drug\;added-Free\;drug}{\mathrm{Total}\;\mathrm{drug}\;\mathrm{added}}\times 100$$

#### 4.7.3. Fourier-Transform Infrared Spectroscopy (FTIR)

An FTIR spectrophotometer was used to investigate the functional groups and molecular interactions within the PTX-S2.2-PBM NPs and HNK-S2.2-PBM NPs. Characteristic peaks corresponding to PTX, HNK, PCL/PEG, and S2.2 aptamer conjugation were identified by recording the Spectra over the 4000–500 cm^−1^ range.

### 4.8. Cell Viability Assay (MTT Assay)

Viability assays were performed for BrCa cells to evaluate the therapeutic potential of the NPs. 5000 MDA-MB-468 and 3000 HCC 70 cells per well were seeded overnight and treated with NPs for 24, 48, or 72 h at 37 °C in a 5% CO_2_ incubator. Prior to use, the NPs were filtered with a sterile 0.22 μm PVDF filter to prevent exposure to contamination. MTT (3-(4,5-dimethylthiazol-2-yl)- 2,5-diphenyltetrazolium bromide; 5 mg/mL) was then added (10 µL) to each well and incubated at 37 °C for 3–4 h. The purple formazan crystals were dissolved in 100 µL of dimethyl sulfoxide (DMSO), and the absorbance was measured at 570 nm with a spectrophotometer.

### 4.9. Live Dead Cell Assay

A live/dead cell viability assay was performed using the Blue/Green ReadyProbes^®^ cell viability imaging kit (Thermo Fisher, Pittsburgh, PA, USA). In short, MDA-MB-468 and HCC 70 cells were cultured at 20,000 cells/well in a 48-well plate. Prior to use, the NPs were filtered with a sterile 0.22 μm PVDF filter to prevent exposure to contamination. After 24 h, HCC70 treatment wells were treated with predetermined IC50 values 6.94 nM PTX-S2.2-NPs and 15.41 μM HNK-S2.2-NPs, while MDA-MB-468 treatment wells were treated with values 11.25 nM PTX-S2.2-NPs and 13.02 μM HNK-S2.2-NPs. The plate was then incubated at 37 °C 5% CO_2_ for 72 h. Following incubation, two drops of NucBlue^®^ Live reagent and NucGreen^®^ Dead reagent per milliliter of media were added to each well and then incubated for 15 min. Subsequently, the cells were imaged using a fluorescence microscope with DAPI and FITC filters. Mean fluorescent intensity was quantified using ImageJ.

### 4.10. Statistical Analysis

Experiments were performed in triplicate, and the results are presented as the mean ± standard deviation. Two-way ANOVA was performed using GraphPad Prism software (version 10.4.1). Results with a *p*-value < 0.05 were deemed statistically significant.

## 5. Conclusions

In conclusion, this study has demonstrated that BrCa tissues exhibit elevated MUC1 expression compared to normal breast tissues, making it a promising target for BrCa-specific therapies. Via in silico studies, we have also shown that PTX and HNK potentially work synergistically when combined to target MUC1. In vitro characterization and cell viability assays ensured the efficacy of the aptamer-conjugated NPs and established PTX-S2.2 and HNK-S2.2 PBM NPs as potentially viable treatment options for MUC1-expressing BrCa. Overall, these findings serve as a basis for future studies to examine further the use of PTX-S2.2 and HNK-S2.2 PBM NPs in combination and their mechanisms of action to treat BrCa effectively.

## Data Availability

This article includes all the data generated and analyzed in the study.

## Supplementary Materials

The following supporting information can be downloaded at: https://www.mdpi.com/article/10.3390/ph18121814/s1. Figure S1: Chou-Talalay isobologram, Figure S2: Expression of MUC-1 in breast cancer cells.

## Abbreviations

The following abbreviations are used in this manuscript:

BrCa	Breast Cancer
PTX	Paclitaxel
HNK	Honokiol
PBM-NPs	Planetary-Balled Milling Nanoparticles
PEG	Polyethylene Glycol
PCL	Polycaprolactone
NHS	N-Hydroxy Succinimide
FBS	Fetal Bovine Serum
RPMI	Roswell Park Memorial Institute
L-15	Leibovitz’s L-15 Medium
DMSO	Dimethyl Sulfoxide
PBS	Phosphate-Buffered Saline
IC50	Half Maximum Inhibitory Concentration
TMA	Tissue Microarray
