# Supplementary material for: In Silico and In Vitro Profiling of Honokiol and Paclitaxel-Loaded PBM Nanoparticles for Targeted Breast Cancer Delivery
Source: Pharmaceuticals (Basel). 2025 Nov 27;18(12):1814. doi: 10.3390/ph18121814 (PMC12735985; doi:10.3390/ph18121814)
Supplement: Supplementary file 1 [file pharmaceuticals-18-01814-s001.zip › pharmaceuticals-3957286-supplementary.pdf]

In Silico and In Vitro Profiling of Honokiol and Paclitaxel-Loaded PBM Nanoparticles for Targeted Breast Cancer Delivery

Briana Kinnel [1,] Amit Kumar Srivastava [1,2], Santosh Kumar Singh [1] and Rajesh Singh [1,3,*]

1 Department of Microbiology, Biochemistry and Immunology, Morehouse School of Medicine, Atlanta, GA 30310, USA

2 School of Forensic Sciences, Uttar Pradesh State Institute of Forensic Sciences, Lucknow 22604, India

3 Cancer Health Equity Institute, Morehouse School of Medicine, Atlanta, GA 30310, USA

* Correspondence: rsingh@msm.edu; Tel.: +1-404-756-6661; Fax: +1-404-752-1179

Supplemental Figures:

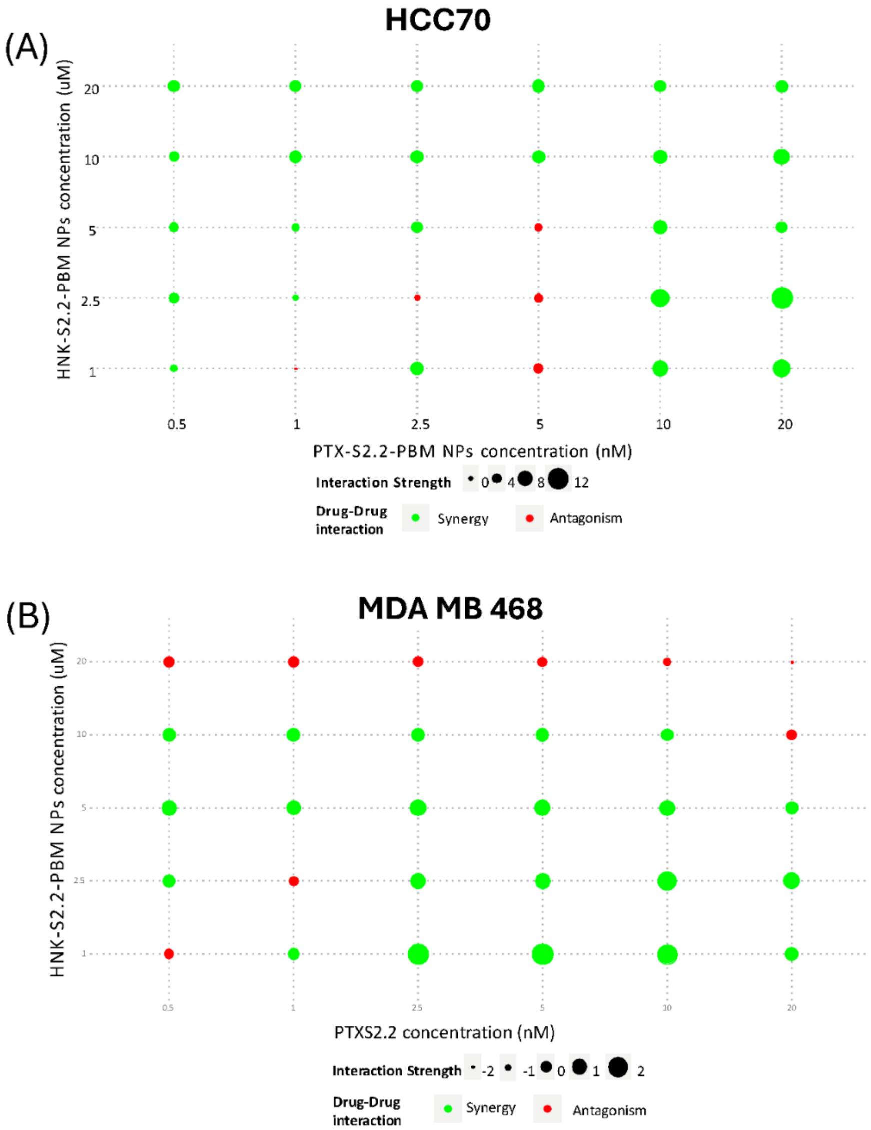

Supplemental Figure S1. Chou-Talalay Isobologram representing drug synergy. Honokiol MUC1 aptamer conjugated NPs (HNK-S2.2-PBM NPs) and Paclitaxel MUC1 aptamer conjugated NPs (PTX-S2.2-PBM NPs) combination drug effect was quantified using MTT and analyzed using Drug-Drug Combination Visualization (DDCV) shiny app using R. (A) HCC 70 Chou–Talalay isobologram. (B) MDA MB 468 Chou-Talalay isobologram.

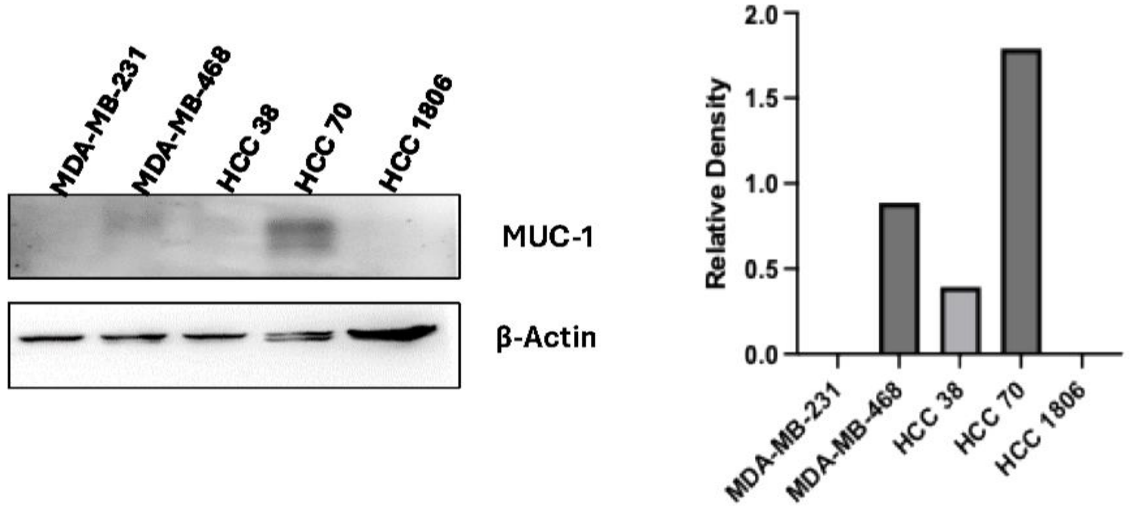

Supplemental Figure S2. Expression of MUC-1 in breast cancer cells. The expression of MUC-1 in breast cancer cell lines (MDA-MB-231, MDA-MB-468, HCC38, HCC70, HCC1806). Immunoblotting was carried out with a primary antibody against the MUC-1 protein (Abcam, 1:1000). As an internal standard for equal loading, blots were probed with an anti-β-Actin antibody.
